# Antiretroviral concentration measurements as an additional tool to manage virologic failure in resource limited settings: a case control study

**DOI:** 10.1186/s12981-019-0255-x

**Published:** 2019-12-06

**Authors:** Allan Buzibye, Joseph Musaazi, Amrei von Braun, Sarah Nanzigu, Christine Sekaggya-Wiltshire, Andrew Kambugu, Jan Fehr, Mohammed Lamorde, Ursula Gutteck, Daniel Muller, Stefanie Sowinski, Steven J. Reynolds, Barbara Castelnuovo

**Affiliations:** 1 Infectious Diseases Institute, Makerere University, College of Health Sciences, P. O. BOX 22418, Kampala, Uganda; 2 Division of Infectious Diseases and Tropical Medicine, University Hospital of Leipzig, University of Leipzig, Leipzig, Germany; 3 Department of Pharmacology, Makerere University, College of Health Sciences, Kampala, Uganda; 4 Department of Public Health, University Hospital, University of Zurich, Zurich, Switzerland; 5 Department of Clinical Chemistry, University Hospital, University of Zurich, Zurich, Switzerland; 6 The Gladstone Institute of Virology and Immunology, University of California, San Francisco, San Francisco, CA USA; 7 Division of Intramural Research, National Institute of Allergy and Infectious Diseases, National Institutes of Health, Bethesda, MD USA; 8 Johns Hopkins University School of Medicine, Baltimore, MD USA

**Keywords:** Virologic failure, Therapeutic drug monitoring, Uganda, Resource limited setting, HIV

## Abstract

**Background:**

Several studies demonstrate a correlation between sub-therapeutic concentrations of antiretroviral drugs and virologic failure. We examined the sensitivity, specificity and predictive values of sub-therapeutic drug levels in predicting viralogic failure.

**Methods:**

This was a case control study with cases being samples of participants with virologic failure, and controls samples of participants with virologic suppression. We analyzed samples obtained from participants that had been on antiretroviral treatment (ART) for at least 6 months. Virologic failure was defined as HIV-RNA viral load ≥ 1000 copies/ml. Sub-therapeutic drug levels were defined according to published reference cutoffs. The diagnostic validity of drug levels for virologic failure was assessed using plasma viral loads as a gold standard.

**Results:**

Sub-therapeutic ART concentrations explained only 38.2% of virologic failure with a probability of experiencing virologic failure of 0.66 in a patient with low drug levels versus 0.25 for participants with measurements within or above the normal range. Approximately 90% of participants with ART concentrations above the lower clinical cut off did not have virologic failure.

**Conclusions:**

These results support prior indication for therapeutic drug monitoring in cases of suspected virologic failure.

## Background

Antiretroviral therapy (ART) has reduced morbidity and mortality in people living with HIV [1, 2]. Currently, the World Health Organization recommends periodic viral load testing for monitoring the effectiveness of ART [3]. Virologic failure is defined according to the 2016 WHO guidelines as two consecutive HIV-RNA viral loads ≥ 1000 copies/ml [3]. Patients with an initial detectable viral load test result receive intensive adherence counselling sessions before the viral load test is repeated, in order to differentiate non-adherence to ART from virologic failure due to drug resistance. When the repeat viral load result remains ≥ 1000 copies/ml patients are switched to another regimen.

Two factors that may contribute to virologic failure include pre-treatment resistance and acquired drug resistance [4, 5]. Acquired drug resistance normally results from sub-therapeutic drug exposure. Factors that may lead to sub-therapeutic exposure include single nucleotide polymorphisms [4, 5], drug–drug interactions, drug–herb interactions, non-adherence and mal-absorption [6–9]. Therapeutic drug monitoring (TDM) may detect suboptimal concentrations, and therefore inform the clinicians on the need to address factors which may lead to drug resistance and prevent unnecessary switching where treatment options are already limited.

Therapeutic drug monitoring is the clinical practice of measuring specific drug levels at designated intervals with the purpose of maintaining a constant blood concentration and optimize dosage for individual patients [10]. Among the antiretroviral drug classes, non-nucleoside reverse transcriptase inhibitors (NNRTIs) and protease inhibitors (PIs) meet the proposed criteria for TDM such as the presence of a dose–response relationship [11]. Several platforms are employed for TDM of HIV drugs [12–14]. In resource-limited settings, high performance liquid chromatography with ultraviolet detection (HPLC-UV) is commonly used. We investigated the sensitivity, specificity and predictive values of NNRTIs and PIs drug levels in predicting virologic failure.

## Materials and methods

This study was conducted at the Infectious Diseases Institute (IDI), Makerere University, an HIV centre of excellence located in Mulago teaching Hospital in Kampala with over 8000 HIV-positive individuals receiving care [15]. The IDI clinic began providing HIV care in 2002, while free ART has been provided since April 2004.

This was a case control study. For our analysis we used samples from patients enrolled in the “Resistance in HIV-infected adults in North and South” (RHINOS) study [16]. The RHINOS study was conducted between June and September 2015 at IDI, and enrolled patients on any first- or second-line ART regimen for at least 6 months. The RHINOS study offered viral load testing to all participants and resistance testing to those with VL > 1000 copies/ml [16].

Cases were defined as participants with virologic failure (VL > 1000 copies/ml), while controls were patients with a measurement of VL ≤ 1000 copies. Controls were matched on age and gender at the ratio of 1:2, and samples were obtained using the cumulative sampling technique.

An in-house multiplex assay high performance liquid chromatography with ultraviolet detection (HPLC-UV) was developed and validated to measure the serum concentration of efavirenz, nevirapine, atazanavir and lopinavir. The HPLC UV system used was a Shimadzu LC-2010HT with an inbuilt auto sampler, pumps, and UV detector (Shimadzu, Kyoto, Japan), controlled by CLASS-VP software version 6.1 (Shimadzu, Kyoto, Japan). The analytical phenyl hexyl column was a Betasil 150 × 3 mm, 5 µm (Thermo Scientific, Waltham, USA) protected by an inline filter. Sample processing involved protein precipitation from serum. Dual detection was achieved at 210 and 254 nm. The mobile phase consisted of 50 mM ammonium acetate/0.1 mmol/l EDTA/0.1% formic acid and acetonitrile/water (9/1 v/v). The flow rate was set at 0.45 ml/min with gradient elution. The method was validated over a concentration range of 1–15 mg/l for nevirapine, efavirenz, lopinavir and 0.2–3 mg/l for atazanavir. The assay was accurate (98.0–109.6%) with inter and intra-day coefficient of variation less than 11%. Drug levels were considered sub-therapeutic if they were below the lower clinical cut-off [17–19].

Serum concentration data were captured using EpiData version 3.1 (EpiData association, Odense, Denmark). Serum data were merged with other data collected from the main RHINOS study dataset and analyzed using STATA/IC version 14.2 (StataCorp, College Station, Texas 77845 USA). McNemar’s Chi-square test was used to compare categorical characteristics across cases and controls, such as WHO stage. Mann–Whitney Wilcoxon rank-sum test was used for continuous variables, like CD4 counts, and serum drug concentrations. Sensitivity, specificity and predictive values (PV) of drug levels in predicting virologic failure was estimated. Receiver operating characteristic (ROC) curve analysis was used to define a cut-off that would provide a maximum value for specificity and sensitivity.

## Results

Of 2511 patients enrolled in RHINOS, 198 (7.9%) patients had VL > 1000 copies/ml, and were eligible as cases; two cases were excluded because they had no matching controls, and five other cases were excluded because samples could not be identified. Therefore, 191 cases and 382 controls for a total of 573 samples were analyzed for serum drug levels.

Table 1 compares the baseline characteristics of the cases and the controls. The median CD4 count was lower in the cases (262 cells/µl,) compared to the controls (500 cells/µl,) (P-value < 0.01). Cases had a higher proportion of participants in WHO 3 or 4 stage, compared to controls (61.8% versus 51.2%, P-value = 0.02). We observed a higher proportion of cases on PI-based ART regimen compared to controls (25.7% versus 11.8%, P-value < 0.01). Cases were matched with controls by gender and age, and therefore, baseline differences were mainly observed in WHO staging and CD4 counts. The average time from the last intake of a dose of ART was 8 h.

**Table 1:** Patients’ characteristics by cases and controls

Characteristics	Cases (VL > 1000) (copies/µl) N = 191	Controls (VL ≤ 1000) (copies/µl) N = 382	P-value
	N (%)	N (%)	
Gender, number (%)			
Male	58 (30.4)	116 (30.4)	–
Female	133 (69.6)	266 (69.6)	–
Age in years, median (IQR)	37 (29−43)	37 (29−43)	–
18–34	75 (39.3)	150 (39.3)	–
≥ 35	116 (60.7)	232 (60.7)	–
WHO stage, number (%)			
1 or 2	73 (38.2)	186 (48.8)	0.02
3 or 4	118 (61.8)	195 (51.2)	
CD4 cell counts per µl, median (IQR)	262 (118−429)	500 (346−661)	< 0.01
Duration on ART in months, median (IQR)	47 (27−82)	46 (27−93)	0.22
Time since HIV diagnosis in years Median (IQR)	7 (3−11)	8 (4−11)	0.30
ART_NNRT (NVP, EFV), number (%)	141 (74.2)	339 (88.7)	–
ART_PI (ATV, LPV), number (%)	49 (25.7)	45 (11.8)	< 0.01

Gender and age were used as matching variables, so P-values on them is not relevant

*N* number, *IQR* interquartile range, *VL* viral load, *WHO* World Health Organization, *ART* antiretroviral therapy, *ATV* atazanavir, *LPV* lopinavir, *EFV* efavirenz, *NVP* nevirapine, *NNRTI* nonnucleoside reverse transcriptase inhibitor, *PI* protease inhibitor

**Fig. 1:**
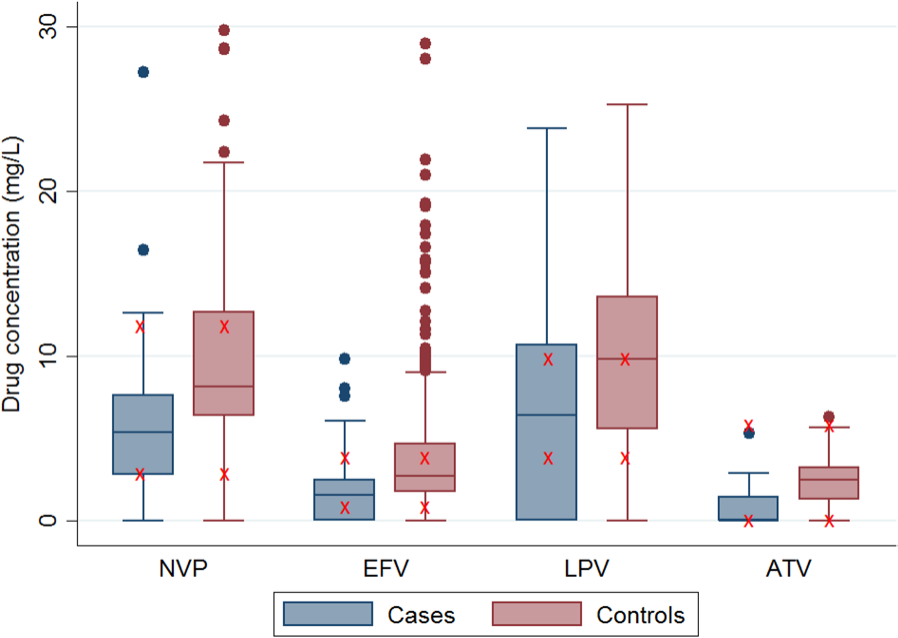
A box plot of drug concentration by drug type stratified by cases and controls. x = Marks the lower and upper cut-off of normal ranges

For nevirapine, efavirenz and atazanavir, mean and the median drug concentrations were significantly lower in the cases compared to controls. For lopinavir a trend towards lower concentrations was noted in cases (Table 2). Most drug levels were within the published clinical ranges for all the drugs (Fig. 1).

**Table 2:** Distribution of drug concentrations by cases and controls

Drug	Cases		Controls		P-value*
	N	Median (IQR)	N	Median (IQR)	
Nevirapine	65	5.37 (2.81–7.64)	116	8.18 (6.38–12.69)	< 0.01
Efavirenz	77	1.60 (0.00–2.50)	221	2.72 (1.78–4.71)	< 0.01
Lopinavir	34	6.41 (0.00–10.70)	24	9.84 (5.54–13.57)	0.06
Atazanavir	15	0.00 (0.00–1.44)	21	2.50 (1.29–3.24)	0.01

*P-values comparing median drug concentrations between cases and controls using Mann–Whitney Wilcoxon rank-sum test. Significant differences were observed in concentrations of nevirapine, efavirenz and atazanavir

Overall, TDM had a sensitivity of 38.2% and a positive predictive value (PPV) of 65.8% for virologic failure. Sensitivity, specificity, PPV and negative predictive value (NPV) respectively for each drug are shown in Table 3. Using ROC curve analysis, for nevirapine, a cut off of 7.8 mg/l resulted in a sensitivity of 78% and a specificity of 56% (AUC-0.71). For efavirenz, a cut off of 2.7 mg/l resulted in a sensitivity of 64% and a specificity of 54% (AUC-0.61). Sample size was inadequate to establish a cut-off for atazanavir. No calculation was performed for lopinavir as there was no significant difference in concentrations between cases and controls.

**Table 3:** SE, SP, PPV and NPV for TDM methods for detecting virologic failure for different ARV drugs

Drug	SE (95% CI)	SP (95% CI)	PPV (95% CI)	NPV (95% CI)
Nevirapine	27.7 (16.8–38.6)	94.0 (89.6–98.3)	72.0 (54.4–89.6)	69.9 (62.7–77.1)
Efavirenz	41.6 (30.6–52.6)	89.1 (85.0–93.2)	57.1 (44.2–70.1)	81.4 (76.5–86.3)
Lopinavir	44.1 (27.4–60.8)	83.3 (68.4–98.2)	78.9 (60.6–97.3)	51.3 (35.6–67.0)
Atazanavir	53.3 (28.1–78.6)	85.7 (70.7–99.0)	72.7 (46.4–99.0)	72.0 (54.4–89.6)
Overall	38.2 (31.3–45.1)	90.1 (87.1–93.1)	65.8 (56.9–74.6)	74.5 (70.5–78.4)

*SE* sensitivity, *SP* specificity, *PPV* positive predictive value, *NPV* negative predictive value

## Discussion

Previous studies have shown a positive relationship between drug levels and virologic failure [19–22]. However, to our knowledge this is the first sub-Saharan Africa study to explore the sensitivity, specificity and predictive values of a panel of non-nucleoside reverse transcriptase inhibitors and protease inhibitors for predicting virologic failure. Serum concentrations of ART among cases were significantly lower than concentrations among controls suggesting that low drug levels contribute to virologic failure. We show that an approach using TDM as a screening tool would identify approximately one third of the cases of virologic failure in our study population.

A patient with a low level of ART was more likely to be experiencing virologic failure compared to a patient with higher concentrations. Generally, the predictive values were 70% or higher except for efavirenz whose PPV was 57.1% and lopinavir whose NPV was 51.3%. Here we report a higher PPV for efavirenz compared to what was reported earlier by Catia and colleagues (50%) [19] probably due to a higher prevalence of virologic failure in our setting compared to the Swiss setting. Our study identified greater sensitivity and specificity when using lower cutoffs for nevirapine (7.8 mg/l) and efavirenz (2.7 mg/l) that were greater than previously published cutoffs (nevirapine 3 mg/l and efavirenz (1 mg/l). Our cut-offs are likely influenced by a greater proportion of participants with high concentrations of NNRTI (Fig. 1) that may be explained by slow metabolizer polymorphisms of cytochrome P450 2B6 that are more common in African settings [23].

Our results confirm previous findings about the performance of TDM in predicting virologic failure when compared with viral load measurements [24]. TDM has been reported to be a poor predictor of virologic failure compared to other adherence measures such as alectronic adherence monitoring device and pharmacy refill [24–26]. However, TDM performs far better than self-reported adherence. In a study done in Cameroon [27], virologic failure was associated with nevirapine concentration but not with self-reported adherence. The sensitivity, specificity, positive and negative predictive values were: 20.5%, 91.7%, 44.4% and 78.0% respectively when TDM was compared to viral loads. The respective values for self-reported adherence were 2.6%, 97.5%, 25.0% and 75.5%.

No reliable data on adherence and time of drug intake was available to support interpretation of the results. It was not possible to determine if the low concentrations were a true reflection of lower steady state concentrations or because some patients had recently had a treatment interruption and the TDM sample was collected while they had not yet achieved steady-state concentration. Prospective studies with serial concentration measurements preceding a virologic outcome would yield additional information on the TDM approach. Our study was conducted in a clinical research facility, where TDM facilities and technical capacity are available and few centers are available for TDM in Africa. In contrast, viral load testing is more readily available in resource limited settings. If TDM were confirmed to provide additional value, feasibility and cost considerations for expansion of this test would need to be explored.

## Conclusion

In agreement with previous studies, this study suggests that low drug levels of ART could contribute to virologic failure. Prospective studies in resource limited settings are needed to investigate this relationship.

## Data Availability

The data that support the findings of this study are available from the RHINOS study team but restrictions apply to availability of these data and so not publicly available. Data are however available from corresponding author upon reasonable request and with permission of the RHINOS study team.

## Abbreviations

ART: antiretroviral therapy
HIV: human immunodeficiency virus
RNA: ribonucleic acid
WHO: World Health Organization
TDM: therapeutic drug monitoring
NNRTIs: nonnucleotide reverse transcriptase inhibitors
P.Is: protease inhibitors
EFV: efavirenz
NVP: nevirapine
LPV: lopinavir
ATV: atazanavir
HPLC-UV: high performance liquid chromatography with ultraviolet detection
VL: viral load
VF: virologic failure
IDI: Infectious Diseases Institute
RHINOS: resistance in HIV-infected adults in North and South
IQR: interquartile range
NVP: negative predictive value
PPV: positive predictive value
SE: sensitivity
SP: specificity
PK: pharmacokinetics
ROC: receiver operating characteristic
